# Emotional urgency predicts bipolar symptoms, severity, and suicide attempt better than non-emotional impulsivity: a cross-sectional study

**DOI:** 10.3389/fpsyg.2023.1277655

**Published:** 2023-12-01

**Authors:** Wen Lin Teh, Jianlin Liu, Nisha Chandwani, Yu Wei Lee, Phern-Chern Tor, Mythily Subramaniam, Roger C. Ho

**Affiliations:** ^1^Yong Loo Lin School of Medicine, National University of Singapore, Singapore, Singapore; ^2^Institute of Mental Health, Singapore, Singapore; ^3^Department of Psychological Medicine, National University of Singapore, Singapore, Singapore

**Keywords:** emotion-based impulsivity, emotional urgency, UPPS-P, trait impulsivity, bipolar disorder

## Abstract

**Introduction:**

Emotional urgency is an emotion-based subdimension of trait impulsivity that is more clinically relevant to psychopathology and disorders of emotion dysfunction than non-emotional subdimensions (i.e., lack of perseverance, sensation seeking, lack of premeditation). However, few studies have examined the relative effects of emotional urgency in bipolar disorder. This cross-sectional study aimed to establish the clinical relevance of emotional urgency in bipolar disorders by (1) explicating clinically relevant correlates of emotional urgency and (2) comparing its effects against non-emotional impulsivity subdimensions.

**Methods and results:**

A total of 150 individuals with bipolar disorder were recruited between October 2021 and January 2023. Zero-order correlations found that emotional urgency had the greatest effect on bipolar symptoms (*r* = 0.37 to 0.44). Multiple two-step hierarchical regression models showed that (1) positive urgency predicted past manic symptomology and dysfunction severity (*b* = 1.94, *p* < 0.001 and 0.35 *p* < 0.05, respectively), (2) negative urgency predicted current depression severity, and (3) non-emotional facets of impulsivity had smaller effects on bipolar symptoms and dysfunction by contrast, and were non-significant factors in the final step of all regression models (*b* < 0.30, ns); Those who had a history of attempted suicide had significantly greater levels of emotional urgency (Cohen’s *d* = –0.63).

**Discussion:**

Notwithstanding the study’s limitations, our findings expand status quo knowledge beyond the perennial relationship between non-emotion-based impulsivity and bipolar disorder and its implications.

## 1 Introduction

Bipolar disorder is described in the Diagnostic and Statistical Manual of Mental Disorders (DSM-IV) as having at least one manic or hypomanic episode comprising abnormally persistent levels of elevated, irritable, expansive mood that severely affects functioning or requires hospitalization (American Psychiatric Association, 2000). The global lifetime prevalence of bipolar disorder, which comprises bipolar I and bipolar II, is approximately 1%, and ranges between 0% and 2.1% across countries (Merikangas et al., 2011; Moreira et al., 2017; Teh et al., 2020).

Bipolar disorder is the 17*^th^* leading cause of disability worldwide, behind depression, anxiety disorders, schizophrenia, and dysthymia (Vigo et al., 2016). Substantial economic burden (Cloutier et al., 2018), poor functioning, cognitive impairment, and premature mortality, especially death by suicide, are typically associated with the disorder (Grande et al., 2016). Therefore, the aim of much of research has been to examine how maladaptive disorder characteristics contribute to poor outcomes, such as disability and low functioning (Lima et al., 2018).

Gray’s biopsychological theory for personality models two biological systems that underlie innate predispositions of avoidance and approach behaviors (Matthews and Gilliland, 1999). According to Gray’s theory, the behavioral activation system (BAS) is a biological framework that underlies the innate drive for approach motivation, and thus provides a biopsychological basis for impulsivity (Matthews and Gilliland, 1999). The BAS dysregulation model thus theorizes that maladaptive regulation of the BAS contributes to manic and depressive states of bipolar spectrum disorders (Depue et al., 1981, 1987; Depue and Iacono, 1989). Impulsivity is one of the several common features of bipolar disorder that is associated with maladaptive behaviors, such as alcoholism or suicidality (Swann, 2009; Creswell et al., 2019). From a neurocognitive perspective, impulsivity is partly attributed by the disruption of cortico-striatal neurocircuitries at neuroanatomical and neurochemical levels (Fineberg et al., 2014; Bora et al., 2019; Lapomarda et al., 2021), which in turn gives rise to suboptimal emotional and stress regulation, decision making, and inhibitory control at the cognitive level (Carvalho et al., 2020).

Conceptually, impulsivity is multifaceted and it can be categorized into two broad and empirically distinct dimensions of state (i.e., behavioral) and trait [i.e., dispositional; (Sharma et al., 2014; Creswell et al., 2019)]. Nevertheless, the latter domain is more widely studied. Trait impulsivity have been operationalized in a myriad of ways, but its core definition remains consistent, and that is the predisposition to react rashly in response to stimuli with little regard for negative (and often long term) consequences (Whiteside and Lynam, 2001; Lynam et al., 2006). Longitudinal research has shown that trait impulsivity has been associated with the onset of the bipolar disorder (Alloy et al., 2012; Saddichha and Schuetz, 2014; Rote et al., 2018); Individuals with bipolar disorder would typically self-rate higher levels of impulsivity than healthy controls; and depending on how impulsivity is operationalized, the extent of the differences ranges from moderate to large (Santana et al., 2022).

According to Whiteside and Lynam (2001), there are five theoretical pathways to impulsive behaviors: (negative) Urgency, the lack of Premeditation, the lack of Perseverance, Sensation seeking, and Positive urgency [UPPS-P; (Cyders and Smith, 2008; Hershberger et al., 2017)]. Negative and positive urgency, hereafter referred to as emotional urgency, represents a unique duo-pathway that entails emotion-caused impulsive behaviors, which is unlike other UPPS-P facets of impulsivity that are construed as non-emotional pathways to rash action (Cyders and Smith, 2008; Cyders et al., 2016). Current findings demonstrate that emotional urgency has a substantial influence on psychopathology (*r* = 0.34) compared to non-emotional forms of impulsivity [*r* ranging from 0.08 to 0.14.; (Berg et al., 2015)].

Research has shown that emotional urgency is highly linked to mania risk in a student cohort sample [*r* = 0.36; (Giovanelli et al., 2013)]. Given that the effect of trait concepts on any given phenomenon is typically small (*r* = 0.19 at the 50*^th^* percentile), and that trait concepts with a moderate effect, if defined by traditional cut-offs, equates to a large effect [*r* = 0.5 at the 75*^th^* percentile, (Gignac and Szodorai, 2016)], emotional urgency is arguably clinically relevant to a large degree and would therefore warrant further scrutiny. Prevailing research suggests that emotion disturbances and maladaptive cognition interact and conjointly contribute to the bipolar experience (Lima et al., 2018). Emotional urgency one particular concept that compounds emotionality and (suboptimal) cognition, yet little is understood of the clinical relevance of this hybrid construct in bipolar disorder. In addition, positive urgency, which is a subfactor of emotional urgency, though intuitively relevant to mania, have not been sufficiently studied in the clinical context. Individual differences in emotion-based impulsivity can potentially indicate worser illness course and/or prognosis since it is particularly associated with externalizing disorders, depression symptomology, and disorders of emotion dysfunction (Berg et al., 2015).

In light of the existing limitations of prevailing research, this study aims to explicate the clinical relevance of emotional urgency by investigating the associations between emotional urgency (negative and positive) and retrospective self-reports of manic symptoms in outpatients diagnosed with bipolar I and II disorders. From the literature review gathered, we hypothesized the following: 1) positive urgency will be associated with mania and its severity, 2) negative urgency will be associated with depression severity, 3) emotional urgency will have the greatest effect on mania than non-emotional facets of impulsivity, and finally, this study will assess the correlates of emotional urgency, such as predominant polarity, psychosis history, suicide attempt, frequency hospitalizations, as these are phenotypes contributing to the clinical heterogeneity of the disorder.

## 2 Materials and methods

### 2.1 Procedure

Participants were referred to the research team by their attending doctors at the outpatient clinic or in the wards at the Institute of Mental Health (IMH) in Singapore. To be eligible, individuals had to be 21–65 years old and were seeking treatment for bipolar disorder as the primary condition. Outpatients and inpatients with bipolar disorder were approached between October 2021 to December 2022. The response rate was 72.5% (150/207). Written informed consent was taken in the presence of a witness. The survey took approximately 1 h to complete, and participants were reimbursed 40 Singapore dollars for their participation. This study was approved by the institution’s internal ethics committee and the Domain Specific Review Board (DSRB; No. 2021/00808) of the National Health Group, Singapore.

### 2.2 Participants

A sample of 150 participants were recruited. The majority of participants were female (50.3%), had bipolar I disorder (89.0%), with tertiary or higher education (40.7%), were of Chinese ethnicity (75.2%), had a manic/hypomanic predominant polarity (47.6%), no history of psychosis (53.1%), and had never attempted suicide (62.8%). Participants were on 38.6 years old on average (SD = 12.0), self-reported age of onset was 25.2 years (SD = 10.8), average lifetime number of hospital visits (with a maximum upper limit of 20) is 4.3 times. The average number of manic symptoms endorsed is 9.4 (SD = 3.48).

### 2.3 Materials

Short Impulsive Behavior Scale (S-UPPS-P): a 20-item scale that measures multi-dimensional impulsivity. The subdomains are negative and positive urgency, lack of perseverance, lack of premeditation, and sensation-seeking; 4-point Likert scale from strongly disagree to strongly agree (Cyders et al., 2014). Items on each subdomain were summed and divided by the total number of items. This scale has good psychometric properties when evaluated in a psychiatric setting (Dugré et al., 2019). The internal consistencies of the subdomains range from 0.68 (lack of perseverance) to 0.84 (positive urgency) in this sample.

Mood Disorder Questionnaire (MDQ): a 15-item self-rated screening scale for bipolar spectrum disorders; It contains 13 items that assess 1) a lifetime history of DSM-IV derived manic/hypomanic symptoms (Yes/No) and 2) 1 item that assesses clinical severity (Hirschfeld et al., 2000); For the last question, participants were asked, “how much of a problem did any of these caused you (e.g., being able to work, having family, money or legal troubles, getting into arguments or fights)?,” where they had to indicate 1 out of 4 responses – “No problem,” “Minor problem,” “Moderate problem,” or “Serious problem.” Higher scores on this item meant lower levels of clinical functioning. The scale has good psychometric properties and contains a moderate overlap with the clinician administered Young Mania Ratings Scale (YMRS; Chrobak et al., 2018). The purpose of using MDQ is to retrospectively document lifetime history of symptoms and clinical severity of individuals already diagnosed with DSM-IV bipolar disorder. The internal consistency score for the MDQ (items 1 to 13) was α = 0.87 in this sample.

Patient Health Questionnaire (PHQ-9): a 9-item major depressive disorder (MDD) module that scores the severity level of depressive symptoms that is compatible with the DSM-IV criteria; it utilizes 4-point Likert scale from “not at all” to “nearly every day” (Löwe et al., 2004). There is good convergent validity between PHQ-9 and clinician-administered Hamilton Depression Rating Scale (HAM-D; Feng et al., 2016). Higher scores indicate higher levels of depression. Internal consistency score for the PHQ-9 was α = 0.91 in this sample.

Altman Self-Rating Mania Scale (ASRM): a short 5-item scale that measures current severity of mania or hypomanic symptoms that is compatible with the DSM-IV criteria; Items include increased talkativeness, elevated mood, decreased need for sleep, high activity levels, increased self-confidence; 5-point Likert scale from “I do not feel more… than usual” to “I feel extremely … all of the time.” The 5-item version of the scale correlates well with other questionnaires of mania, such as the Clinician-Administered Rating Scale for Mania (Altman et al., 2001). The internal consistency score for ASRM was α = 0.75 in this sample.

An additional set of questions that asked of sociodemographic information, such as current age, gender, highest education level, ethnicity; bipolar disorder type (Bipolar I, Bipolar II, or other), age of onset, illness predominant polarity (i.e., majority of episodes being characterized as depressive or manic/hypomanic), history of psychotic features (responses: Yes/No), number of hospitalizations (with a maximum response of “20 or more”), suicide attempt (responses: Yes/No). Five “trap” questions were inserted throughout the questionnaires to identify patterned/careless responses. Participants were directly instructed to circle a response, i.e., “please circle this item as …” and those who responded incorrectly to the “trap” items had their responses for the entire scale removed from analyses.

### 2.4 Statistical analysis

G*power software was used to make *a priori* power calculations for linear multiple regression tests (Faul et al., 2007). A sample size of 147 was required to attain a power of 0.90, a medium effect size of f^2^ 0.15, alpha value of 0.05, with 10 predictors (assumed equal magnitude of effects).

Descriptive statistics were conducted to ascertain the characteristics of the sample. Pearson and point biserial correlation analyses were conducted to ascertain the correlations between all dimensions of UPPS-P trait impulsivity and various clinical parameters (e.g., depression and manic symptom severity). Multiple independent sample *t*-tests were conducted to investigate if group differences in impulsivity scores were present among sub-groups that were differentiated by suicide history, psychosis history, and predominant polarity. To minimize type I error that may occur from conducting 18 independent *t*-tests, a Bonferroni adjusted *p*-value of 0.0028 was used as the cut-off for statistical significance. Multiple hierarchical regression analyses were conducted to investigate if emotonal urgency was associated with bipolar symptoms and severity. Bipolar disorder symptoms and severity were (separately) regressed on sociodemographic factors (i.e., age, sex, ethnicity, highest education) and other UPPS-P subdomains (i.e., lack of perseverance, lack of premeditation, sensation seeking) were entered in the initial step, and positive and negative urgency were entered in the second step. To improve statistical power, cases with less than or equal to 5% missing values at the domain level were replaced by the item’s mean values (rounded to the nearest whole number); cases with greater than 5% missing were removed entirely from the analyses by listwise deletion; Data from five participants were additionally removed due to pattern or problematic responses (i.e., incorrect responses to “trap” questions, e.g., “please circle “3” for this item”). All analyses were conducted in R and SPSS.

## 3 Results

### 3.1 Correlations between UPPS-P subdomains

Correlational analyses of the UPPS-P domains revealed that negative and positive urgency were highly correlated whereas emotional urgency had low to moderate correlations with the remaining three UPPS-P domains. Lack of perseverance was least associated with emotional urgency but remained moderately associated with the lack of premeditation and sensation seeking. All zero-order correlations are summarized in Tables 1, 2.

**TABLE 1:** Zero-order correlation between UPPS-P dimensions of impulsivity, current depression levels, mania severity levels, history of psychosis, suicide attempt, age of onset, hospitalization frequency.

	No. of bipolar symptoms	Clinical severity of symptoms	Curr. depression severity	Curr. mania severity	Hx of psychosis (N/Y)	Suicide attempt (N/Y)	Age of onset	Hosp. freq
Urgency	0.44**	0.44**	0.37**	0.11	0.23**	0.29**	−0.22**	0.19*
Neg Urg	0.36**	0.40**	0.41**	0.06	0.2*	0.32**	−0.22*	0.16
Pos Urg	0.44**	0.40**	0.27**	0.14	0.21*	0.22**	−0.19*	0.19*
Sen. Seek	0.14	0.03	0.09	0.21**	0.16	−0.01	−0.09	−0.01
Premeditation	0.30**	0.27**	0.23**	−0.18*	0.09	0.19*	−0.11	−0.01
Perseverance	0.12	0.05	0.04	−0.23**	−0.02	−0.12	0.00	−0.11
General Impulsivity	0.45**	0.38**	0.34**	0.03	0.22*	0.21*	−0.20*	0.09

**Correlation is significant at the 0.01 level (2-tailed); *Correlation is significant at the 0.05 level (2-tailed); r is Pearson correlation coefficient; Urgency or emotional urgency is the amalgamation of negative (Neg Urg) and positive urgency (Pos Urg); Sen. Seek is Sensation Seeking; Premed is Lack of Premeditation; Persev is Lack of Perseverance; the number of and clinical severity of bipolar symptoms on functioning (item 15) are measured by the Mood Disorder Questionnaire (MDQ); Higher scores on MDQ item 15 denote lower levels of clinical functioning; General impulsivity is the summation of all subdomains; Curr means Current; Hx means History; suicide attempts are dichotomous (No/Yes) variables; Hosp. Freq variable means number of hospitalizations and it is treated as a continuous variable with an upper limit of “20 or more”; point-biserial correlation were conducted between dimensions of impulsivity and dichotomous variables.

**TABLE 2:** Zero-order correlation between UPPS-P subdimensions.

		Mean	SD	1	2	3	4	5	6
1	Emotional Urgency	4.87	1.25	–					
2	Negative Urgency	2.49	0.66	–	–				
3	Positive Urgency	2.38	0.69	–	0.68**	–			
4	Sensation Seeking	2.36	0.61	0.28	0.19*	0.32**	–		
5	Lack of Premeditation	1.96	0.54	0.4**	0.41**	0.32**	0.08	–	
6	Lack of Perseverance	2.00	0.52	0.03	0.09	−0.04	−0.19*	0.48**	–

**Correlation is significant at the 0.01 level (2-tailed); *Correlation is significant at the 0.05 level (2-tailed); *r* is Pearson correlation coefficient; Emotional urgency is negative and positive urgency.

### 3.2 Group differences in suicide attempts, predominant polarity, history of psychosis

The results are summarized in Table 3. There was a significant group-difference in emotional urgency and negative urgency scores between those with and without a history of attempted suicide (*p* < 0.001; *p* = 0.009 for positive urgency, *ns*). No significant between group differences were found for non-emotional dimensions of impulsivity for all indicators (*p* > 0.02, *ns*). There was no significant group differences in impulsivity found for history of psychosis or manic/depressive polarity.

**TABLE 3:** Subgroup differences in all subdomains of impulsivity (S-UPPSP), *n* = 145.

	Emotional urgency								Negative urgency							Positive urgency						
	*n*	M	SD	*t*	df	*d*	95% CI LL	95% CI UL	M	SD	*t*	df	*d*	95% CI LL	95% CI UL	M	SD	*t*	df	*d*	95% CI LL	95% CI UL
**Predominant Polarity**																						
Manic/Hypomanic	69	2.38	0.64	−0.98	128	−0.17	−0.31	0.11	2.36	0.65	−2.31	128	−0.40	−0.47	−0.04	2.40	0.71	0.38	128	0.07	−0.19	0.28
Depressed	64	2.48	0.55						2.61	0.59						2.35	0.66					
**History of psychosis**																						
Absent	77	2.28	0.59	−2.76	139	−0.46	−0.48	−0.079	2.34	0.65	−2.44	139	−0.41	−0.48	−0.05	2.23	0.66	−2.59	139	−0.42	−0.53	−0.07
Present	67	2.56	0.62						2.60	0.62						2.52	0.71					
**Suicide attempt history**																						
Never	91	2.28	0.60	−**3.59**	**140**	−**0.63**	−**0.57**	−**0.17**	2.31	0.67	−**3.98**	**140**	−**0.72**	−**0.64**	−**0.22**	2.23	0.67	−**2.64**	**140**	−**0.44**	−**0.55**	−**0.08**
Ever Attempted	54	2.65	0.57						2.74	0.51						2.53	0.70					
	**Sensation seeking**								**Lack of perseverance**							**Lack of premeditation**						
	***n***	**M**	**SD**	***t***	**df**	***d***	**95% CI LL**	**95% CI UL**	**M**	**SD**	***t***	**df**	***d***	**95% CI LL**	**95% CI UL**	**M**	**SD**	***t***	**df**	***d***	**95% CI LL**	**95% CI UL**
**Predominant polarity**																						
Manic/Hypomanic	69	2.40	0.55	0.61	128	0.11	−0.16	0.30	2.03	0.53	0.76	128	0.13	−0.11	0.25	1.93	0.47	−0.47	128	−0.07	−0.23	0.14
Depressed	64	2.33	0.75						1.96	0.51						1.97	0.62					
**History of psychosis**																						
Absent	77	2.27	0.61	−1.91	139	−0.32	−0.41	0.01	2.00	0.49	0.21	139	0.04	−0.16	0.19	1.90	0.53	−1.02	139	−0.19	−0.27	0.09
Present	67	2.47	0.65						1.98	0.57						2.00	0.55					
**Suicide attempt history**																						
Never	91	2.36	0.62	0.07	140	0.00	−0.21	0.23	2.04	0.54	1.38	140	0.25	−0.05	0.30	1.86	0.55	−2.30	140	−0.42	−0.40	−0.03
Ever Attempted	54	2.36	0.67						1.91	0.49						2.08	0.49					

M is mean, SD is standard deviation, n is group sample size, d is cohen’s effect size, 95% CI is 95% confidence interval of group differences (lower and upper limits); t is the t score determined by independent t-test; predominant polarity refers to the self-reported nature of majority of mood episodes experienced; History of psychosis and suicide attempt history are determined by self-report; bold values indicate significant at *p* < 0.0028 (bonferroni adjusted = 0.05/18 tests).

### 3.3 Association with bipolar symptoms, severity, impact on clinical functioning

Univariate and multiple hierarchical regression analyses were conducted to ascertain the association between emotional urgency and mania symptoms, and separately for depression severity, before and after accounting for extraneous variables. The results are summarized across Tables 4–6. Negative and positive urgency were independently associated with the number of manic symptoms retrospectively reported, *F*(1,134) = 21.1, *p* < 0.001 and *F*(1,134) = 35.4, *p* < 0.001 respectively. However, only positive urgency remained a significant factor after covariates were taken into account in the hierarchical model, *F*(15,120) = 5.4, *p* < 0.001, *R*^2^change = 0.10 (Table 4). Similarly, negative and positive urgency were each independently associated with current depression severity scores, *F*(1,134) = 25.73, *p* < 0.001 and *F*(1,134) = 9.62, *p* = 0.002, respectively; only negative urgency remained a significant factor in the multivariate model, *F*(14,121) = 3.16, *p* < 0.001, *R*^2^change = 0.1 (Table 6).

**TABLE 4:** Univariate and hierarchical linear regression of emotional urgency predictors and lifetime history of manic symptoms (MDQ).

	Univariate					Multiple hierarchical regression									
						Model 1					Model 2				
Variable	b	SE	t	95%CI LL	95%CI UL	b	SE	t	95%CI LL	95%CI UL	b	SE	t	95%CI LL	95%CI UL
Perseverance						0.28	0.62	0.45	−0.96	1.52	0.47	0.59	0.81	−0.69	1.64
Premeditation						1.45*	0.61	2.39	0.25	2.66	0.79	0.60	1.31	−0.40	1.99
Sensation Seeking						0.15	0.48	0.31	−0.80	1.09	−0.35	0.46	−0.76	−1.27	0.57
Negative Urgency	1.98***	0.43	4.59	1.12	2.83						−0.15	0.59	−0.25	−1.32	1.02
Positive Urgency	2.26***	0.38	5.94	1.51	3.02						1.94	0.52***	3.71	0.91	2.97
	**Negative Urgency**			**Positive Urgency**		**Model 1**					**Model 2**				
R-sq	0.136			0.21		0.31					0.40				
ΔR-sq											0.10				
ΔF	21.1			35.4		4.13					9.76				
*p*	<0.001			<0.001		<0.001					<0.001				

Outcome variable is the lifetime history of manic symptoms endorsed measured by the MDQ; b is the unstandardized beta coefficient; t is the t-score; SE means standard error; 95% CI is the 95% confidence interval (lower and upper limits); Variable level indicated in brackets () indicate reference group; the univariate column represents linear regression results where MDQ is regressed on to negative or positive urgency in separate univariate models (1 IV → 1 DV); the “multiple hierarchical regression” columns represent results of analyses of step 1 that included covariates of age, age of onset, sex, highest education, ethnicity, disorder type (bipolar I or II), current mania and depression severity (model 1), and step 2 involving negative and positive urgency (model 2) of the hierarchical regression analysis; R-sq indicates R-squared; Δ denotes change; *,**,*** denote significant at *p* < 0.05, *p* < 0.01, and *p* < 0.001, respectively.

**TABLE 5:** Univariate and hierarchical linear regression of emotional urgency predictors and clinical severity (MDQ).

	Univariate					Multiple hierarchical regression									
						Model 1					Model 2				
Variable	b	SE	t	95%CI LL	95%CI UL	b	SE	t	95%CI LL	95%CI UL	b	SE	t	95%CI LL	95%CI UL
Perseverance						−0.20	0.19	−1.10	−0.57	0.16	−0.18	0.18	−1.02	−0.53	0.17
Premeditation						0.41*	0.18	2.24	0.05	0.77	0.18	0.18	1.01	−0.18	0.54
Sensation Seeking						−0.08	0.14	−0.54	−0.36	0.20	−0.18	0.14	−1.32	−0.46	0.09
Negative Urgency	0.58***	0.11	5.09	0.35	0.80						0.23	0.17	1.35	−0.11	0.58
Positive Urgency	0.52***	0.11	4.97	0.32	0.73						0.35*	0.15	2.27	0.04	0.66
	**Negative urgency**			**Positive urgency**		**Model 1**					**Model 2**				
R-sq	0.16			0.156		0.19					0.28				
ΔR-sq											0.095				
ΔF	26.176			24.773		2.20					7.96				
*p*	<0.001			<0.001		0.013					<0.001				

Outcome variable is self-perceived severity of dysfunction caused by past manic symptoms measured by the MDQ; b is the unstandardized beta coefficient; t is the t-score; SE means standard error; 95% CI is the 95% confidence interval (lower and upper limits); Variable level indicated in brackets () indicate reference group; the univariate column represents linear regression results where MDQ is regressed on to negative or positive urgency in separate univariate models (1 IV → 1 DV); the “multiple hierarchical regression” columns represent results of analyses of step 1 that included covariates of age, age of onset, sex, highest education, ethnicity, disorder type (bipolar I or II), current mania and depression severity (model 1), and step 2 involving negative and positive urgency (model 2) of the hierarchical regression analysis; R-sq indicates R-squared; Δ denotes change; *,**,*** denote significant at *p* < 0.05, *p* < 0.01, and *p* < 0.001, respectively.

**TABLE 6:** Univariate and hierarchical linear regression of emotional urgency predictors on current depression severity (PHQ9).

	Univariate					Multiple hierarchical regression									
						Model 1					Model 2				
Variable	b	SE	t	95%CI LL	95%CI UL	b	SE	t	95%CI LL	95%CI UL	b	SE	t	95%CI LL	95%CI UL
Perseverance						−0.75	1.31	−0.43	−3.33	1.84	−0.63	1.24	−0.50	−3.09	1.83
Premeditation						2.64*	1.25	2.12	0.17	5.12	0.75	1.28	0.59	−1.78	3.28
Sensation seeking						0.36	1.00	0.36	−1.62	2.34	0.03	0.98	0.03	−1.91	1.98
Negative urgency	4.15***	0.82	5.07	2.53	5.76						3.50**	1.21	2.89	1.11	5.91
Positive urgency	2.46***	0.79	3.1	0.89	4.04						0.44	1.11	0.40	−1.75	2.63
	**Negative urgency**			**Positive urgency**		**Model 1**					**Model 2**				
R-sq	0.161			0.066		0.17					0.27				
ΔR-sq											0.10				
ΔF	25.734			9.6153		2.10					8.05				
*p*	<0.001			<0.01		<0.05					<0.001				

Outcome variable is current depression severity measured by PHQ9; b is the unstandardized beta coefficient; t is the t-score; SE means standard error; 95% CI is the 95% confidence interval (lower and upper limits); Variable level indicated in brackets () indicate reference group; the univariate column represents linear regression results where MDQ is regressed on to negative or positive urgency in separate univariate models (1 IV → 1 DV); the “multiple hierarchical regression” columns represent results of analyses of step 1 that included covariates of age, age of onset, sex, highest education, ethnicity, disorder type (bipolar I or II), current mania and depression severity (model 1), and step 2 involving negative and positive urgency (model 2) of the hierarchical regression analysis; R-sq indicates R-squared; Δ denotes change; *,**,*** denote significant at *p* < 0.05, *p* < 0.01, and *p* < 0.001, respectively.

As *current* mania severity and emotional urgency were not significantly correlated (reported in Table 1), the pairing was not analyzed further. Instead, the association between emotional urgency and clinical functioning measured by the MDQ was analyzed. Patterns of associations were similar here at the univariate level of negative and positive urgency, *F*(1,134) = 26.18, *p* < 0.001 and *F*(1,134) = 24.8, *p* < 0.00 respectively. Multiple hierarchical regression showed that only positive urgency was associated with the clinical functioning, albeit to the smaller degree, *F*(15,120) = 3.19, *p* < 0.001, *R*^2^change = 0.1 (Table 5). For all three sets of univariate and multivariate analyses, negative and positive urgency accounted for approximately 10% of all model variances.

## 4 Discussion

The study aimed to explicate the clinical significance and relevance of emotional urgency in bipolar disorder by examining the associations between emotional urgency, bipolar symptoms, and clinically relevant indicators. Two of three of our hypotheses were supported. That is, 1) emotional urgency had the greatest effect on mania, dysfunction severity, and present depressive severity than other subdomains of impulsivity; 2) After various factors were considered, positive but not negative urgency was significantly associated with the endorsement of past manic symptoms and greater levels of symptom dysfunction, whereas negative but not positive urgency was significantly associated with current severity of current depression; 3) it was hypothesized that positive urgency would significantly predict clinical severity of mania, but this was only partially supported—while positive urgency had a small, non-significant effect on current severity, it had a moderate effect on the overall dysfunction severity of mania.

A meta-analytic report showed that non-emotional non-planning impulsivity is a key sub-trait associated with bipolar disorder (Saddichha and Schuetz, 2014). Results of this study was consistent with this finding, as shown in the first step of the multiple hierarchical regression models in Tables 4–6, but we were able to extend this finding further by demonstrating that emotional urgency may be a crucial confound. That is, when emotional urgency was added to the last step of hierarchical regression analyses, the lack of premeditation was reduced to a non-significant factor. Past research has shown that affective dysfunction is contributed primarily by maladaptive emotion-cognition interactions (Muhtadie et al., 2014; Berg et al., 2015; Lima et al., 2018). This study adds new evidence indicating that trait concepts involving emotion-cognition synergy, such as emotional urgency (i.e., the tendency to respond with rash action during emotionally ladened contexts), may have a larger effect on affective dysfunction than non-emotional subdimensions (e.g., a lack of premeditation). However, past studies have focused almost exclusively on non-emotional facets of impulsivity (Newman and Meyer, 2014; Saddichha and Schuetz, 2014; Ramírez-Martín et al., 2020). The current results and past research (Berg et al., 2015) conjointly indicate that it is indeed crucial to consider emotion-based impulsivity in all future research examining trait impulsivity in affective disorders.

Among all subdimensions of impulsivity, positive urgency was most clinically relevant to retrospective reports of mania (*r* = 0.44). The combined dimension of emotional urgency had a similar effect. In addition, given that the correlation between positive and negative urgency is high at *r* = 0.68, it suggests that the separation of subdomains by valence may be unnecessary. However, results from *t*-tests and regression showed that negative and positive urgency were independently associated with different clinical outcomes. For instance, negative urgency differentiated those who never attempted suicide against those who had by a large effect (i.e., effect size *d* of 0.72), but not positive urgency. Thus, current data suggests that valence distinction is clinically relevant and should be retained where necessary.

Emotional urgency was not correlated with current mania severity and while this result is unexpected, it can be explained *post hoc* by the understanding that ASRM does not overtly measure behavioral dysfunction at face value (e.g., “I am constantly active on the go all the time”). Therefore, while a small non-significant effect of positive urgency was found for ASRM (*r* = 0.11, ns)—which is similar to that found by Giovanelli et al. (*r* = 0.13, *p* < 0.01; (Giovanelli et al., 2013)—this study was insufficiently powered to detect significance for a small effect. On the contrary, as emotional urgency is more closely tied to the behavioral domain— its moderate effect on dysfunctional behavioral symptoms captured by the MDQ is thus expected (e.g., “you did things that were unusual for you or that other people might have thought were excessive, foolish, or risky?”). Furthermore, it may be argued that emotional urgency *is* a feature of mania, and thus the relationship between the two is inflated, however, low to moderate zero-order correlations found between emotional urgency and manic symptoms (measured by MDQ) suggested otherwise—these constructs are not multicollinear and are statistically more distinct than similar (*r* = 0.11 to 0.44). The results thus demonstrated unplanned support for discriminant validity for emotional urgency as a construct of the trait tendency for impulsive *behaviors*.

Similar investigations have been conducted in the United States and Canada (Quilty et al., 2010; Victor et al., 2011; Muhtadie et al., 2014; Johnson and Carver, 2016; Johnson et al., 2017; Reich et al., 2019; Shakeel et al., 2019), and to the best of knowledge, this is the first study conducted in a non-western and multi-ethnic community in Southeast Asia. Our results are in line with past research which indicated that emotional urgency is most highly associated with mood severity relative to other non-emotional subdomains (Muhtadie et al., 2014; Berg et al., 2015). Similar to a past report (Johnson and Carver, 2016), the relationship between positive urgency and *current* manic symptom severity was not significant, in part, due to statistical reasons described previously.

The risk of suicide mortality in bipolar disorder is the second most severe after schizophrenia spectrum disorders (Yeh et al., 2019) and while the ratio of suicide attempts to suicide deaths is higher in bipolar disorder (3:1) than in the community (35:1; Plans et al., 2019), the contribution of manic symptoms, anxiety, severe depression at explaining suicidality were, at best, marginal (Persons et al., 2022). Consequently, while pharmacotherapy has been efficacious in reducing depressive symptoms in individuals with bipolar disorder, its efficacy at reducing suicidality remains limited (Silva et al., 2013; Levenberg and Cordner, 2022). The present findings replicate prior research regarding the association between emotional urgency and self-harm/suicidality (Johnson et al., 2017; You et al., 2020). Given that Bipolar disorder has a high degree of heterogeneity in clinical presentation where different phenotypes typically indicate differential prognosis and treatment responses (Alda, 2004; Alda et al., 2009; Benvenuti et al., 2015; Coombes et al., 2020). Greater scores on emotional urgency may thus indicate differential illness course related to suicidality (Colom et al., 2006; Bora et al., 2010).

### 4.1 Clinical implications and future research

A key strategy for addressing clinical heterogeneity is to identify individual differences and clinical phenotypes (Hasler et al., 2006; Guglielmo et al., 2021). This study has shown that emotional urgency may be a crucial candidate phenotype that is associated with adverse indicators. Our results suggest that mental health professionals should assess patients’ narratives of impulsivity under emotionally charged situations. Impulsive behaviors committed under heightened positive emotions may be indicative of severe mania, whereas impulsivity presented in heightened negative emotions may be indicative of an increased risk of suicide.

Psychotherapy for bipolar disorder typically involves cognitive behavioral therapy (CBT), psychoeducation, Interpersonal and Social Rhythm Therapy (IPSRT), among others (Nusslock et al., 2009; Reinares et al., 2014). CBT and psychoeducation have shown to be most effective (than treatment as usual) at stabilizing manic symptoms (Miklowitz et al., 2021). However, these therapeutic interventions generally do not address regulation difficulties or BAS hypersensitivity that arise during heightened emotional contexts (Nusslock et al., 2009). While CBT teaches attention to heightened goal-driven thoughts and destabilizing behaviors to curb impulsivity (Depp et al., 2022), our results indicate that bringing awareness to heightened positive emotions that drive rash thoughts and decisions may be more relevant for bipolar disorder.

### 4.2 Strengths and limitations

This study has certain limitations that warrant acknowledgment. Firstly, due to the cross-sectional nature and convenient sampling of data, causal and temporal connections cannot be derived. Furthermore, the reliance on retrospective reports introduces the possibility of bias, as these responses were not cross-referenced with medical records. Secondly, given that the results are based entirely on self-report data, readers should exercise caution when interpreting the findings. Subsequent research is needed to replicate the findings in order to evaluate their reliability. Thirdly, results may not be representative of non-responders or those who sought treatment outside of the hospital. Fourthly, due to resource constraints, this study was unable to include clinician administered questionnaires to assess current depression and manic severity, which are gold standards of assessments. Thus, the findings may be over- or underestimated due to bias.

Nonetheless, these limitations are mitigated by the strengths of this study. Notably, a high participation response rate was achieved, and a large bipolar patient participant sample was drawn from IMH, which is the sole tertiary psychiatric hospital in Singapore. Another noteworthy aspect is that sociodemographic factors, including age of onset, and non-emotional facets of impulsivity were accounted for in all regression analyses. Furthermore, a stringent *p*-value threshold was applied to mitigate type I error for all between-group analyses.

## 5 Conclusion

Notwithstanding existing limitations, this study has shown that emotional urgency had the greatest effect on retrospective reports of mania and current depression. Additionally, emotional urgency can better explain bipolar heterogeneity than non-emotional facets of impulsivity.

## Data availability statement

The raw data supporting the conclusions of this article will be made available by the authors, upon reasonable request.

## Ethics statement

The studies involving humans were approved by the Domain Specific Review Board from the National Health Group Singapore. The studies were conducted in accordance with the local legislation and institutional requirements. The participants provided their written informed consent to participate in this study.

## Author contributions

WT: Conceptualization, Data curation, Formal analysis, Funding acquisition, Investigation, Methodology, Writing – original draft, Writing – review and editing. JL: Data curation, Methodology, Writing – review and editing. NC: Investigation, Writing – review and editing. YL: Investigation, Writing – review and editing. P-CT: Investigation, Writing – review and editing. MS: Resources, Supervision, Writing – review and editing. RH: Resources, Supervision, Writing – review and editing.
